# Phytogenic Blend Improves Intestinal Health and Reduces Obesity, Diabetes, Cholesterol and Cancers: A Path toward Customised Supplementation

**DOI:** 10.3390/antibiotics11101428

**Published:** 2022-10-18

**Authors:** Sung J. Yu, Yadav S. Bajagai, Friedrich Petranyi, Sara de las Heras-Saldana, Thi Thu Hao Van, Dragana Stanley

**Affiliations:** 1 Institute for Future Farming Systems, Central Queensland University, Rockhampton, QLD 4702, Australia; 2 Animal Genetics and Breeding Unit, University of New England, Armidale, NSW 2351, Australia; 3 School of Science, RMIT University, Bundoora West Campus, Bundoora, VIC 3083, Australia

**Keywords:** essential oils, antibiotics, cancer, lipid, cholesterol, phytogenic, RNAseq

## Abstract

Poultry production is among the most challenging industries for pathogen control. High animal density and abundance of faecal material demand strict biosecurity measures and continual vigilance in monitoring animal health parameters. Despite this vigilance, dealing with disease outbreaks is a part of farmers’ routines. Phytogenic feed additives comprised of herbs, spices, essential oils, and oleoresins have potent antimicrobial and anti-inflammatory actions. Related studies are gaining substantial interest in human and animal health worldwide. In this study, a commercial blend phytogenic feed additive was supplemented to layers in an industrial free-range production system with 20,000 birds in both control and treatment groups. At the end of the trial, the ileum tissue was sampled for RNAseq transcriptomic analysis to study the host reaction to the supplement. Phytogenic supplement significantly inhibited four cholesterol-related pathways and reduced the Arteriosclerosis disease category towards improved cardiovascular health. The supplemented birds exhibited reduced disease susceptibility for 26 cancer categories with *p*-values in the range from 5.23 × 10^−4^ to 1.02 × 10^−25^. Major metabolic shifts in Lipid metabolism in combination with Carbohydrate metabolism have resulted in a decrease in the Obesity category, altering the ratio of fat and carbohydrate metabolism toward lower fat storage.

## 1. Introduction

For decades, public awareness of the cross-resistance of pathogens to antibiotics had gradually increased and initiated a ban on in-feed antibiotic growth promoters (AGPs) in the livestock and poultry industry in many countries [1]. Restricting the use of antibiotics has brought an upsurge in the research focusing on alternative antimicrobial supplements and improved management and biosecurity methods to maintain productivity and welfare in the absence of antibiotics. Eliminating AGPs can potentially aggravate the danger of bacterial diseases and intestinal “dysbiosis” in birds [2]. Understanding the importance of the interplay between the host and gut microbiota is essential to achieve optimum gut microbiota balance and overall health [3].

Hosts’ constant interaction with the beneficial microbiota in the gut plays a critical role in birds’ growth and health [4,5]. There are numerous factors known to modify the intestinal microbiota profile, such as the nutrition [6,7], environmental factors [8,9], and age [10]. Among these, nutrition is a key factor influencing gut microbiota [5]. Plant-based antimicrobials often found in essential oils, also referred to as phytogenic feed additives (PFA), are being actively investigated as potential alternatives to antibiotics and used to improve birds’ welfare and mitigate antimicrobial resistance.

Modern molecular techniques, such as RNA sequencing (RNA-seq), are becoming vital for investigating PFA’s general mechanism in the hosts’ organ health and disease predisposition. The ileum is in direct contact with altered microbiota and the supplemented PFA and is thus suitable tissue to investigate the transcriptomic impact of feed additives on the host gene transcription [11]. This region of the intestine is essential for pathogen infiltration and continually exposed to antigens and immunomodulatory agents from foods and bacteria [12].

Studies investigating transcriptomic effects of the PFA supplementation in birds reported elevated expression of antioxidant genes [13], increased endocytosis and autophagy [14], inhibition of steroid hormones [15], and an increase in fat and carbohydrate metabolism-related pathways [16]. The outcomes of these studies indicated a high level of PFA interference with disease and metabolism-related pathways and interference with the efficiency of numerous medical drugs and supplements [15].

In vitro studies have highlighted that PFAs have numerous benefits, including antifungal, antiviral, antibacterial, antimycotic, antiparasitic, anti-inflammatory, antioxidant, antitumor, and anti-toxigenic properties [17,18,19]. The antioxidant effects have several health benefits [20] by minimising the toxic effects of reactive oxygen species (ROS) in cells. Strong antioxidant activity was observed in the mixture of thymol and carvacrol [21,22]. This property possessed by some PFAs has greatly increased the levels of superoxide dismutase, glutathione peroxidase, and polyunsaturated fatty acids in the tissue [23], which delayed lipid oxidation, enhanced birds’ performance and improved digestive enzymes and immune response [18]. These beneficial properties are related to the course of physiological reactions driven by the enzymes, hormones, and metabolic changes in cells, hence improving the health and performance of birds [24]. PFA-induced improvements included higher quality of the eggs produced, reduced mortality, reduced feed conversion ratio (FCR), and an increase in weight gain [25,26,27,28].

Here, we investigated the effects of a commercial PFA blend comprised of a mixture of essential oils from the *Myrtaceae* and *Asteraceae* plant families and saponins supplemented to commercial free-range layer birds. We have recently reported performance and microbiome, including functional metagenomic alterations, in the present study [28]. Birds supplemented with PFA had reduced mortality and lower number of dirty eggs, increased rate of lay, and increased average egg weight [28]. These performance benefits were particularly evident during a natural Spotty Liver Disease outbreak in these sheds. Functional analysis established a considerable functional alteration in the microbial community, with PFA reducing a range of microbial functions, including the production of essential vitamins, cofactors, energy, and amino acids [28]. PFA supplementation induced a phenotypic shift in the intestinal bacterial lipopolysaccharide (LPS) phenotype towards the less pathogenic form [28].

To our knowledge, this is the first ileum gene expression study based on this phytogenic blend in layer birds. A similar ileum transcriptomic study on the effects of different blends of phytogens has reported an increase in the range of beneficial roles such as vasculogenesis, homeostasis, engulfment of tumour cells, the function of muscle, autophagy, obesity, gut health immune system and much more [14]. Industry-based trials have less control over environmental and biosecurity conditions and usually come with a very high number of birds per shed, up to 100,000 birds, which causes issues with sample size. However, they provide insight into various stresses, natural disease outbreaks, and environmental extremes that could never be replicated in fully controlled research facilities.

In this manuscript, we will continue this investigation to examine the effect of prolonged use of this PFA blend on the ileum transcriptome and evaluate possible mechanisms contributing to reduced mortality, reduced diarrhoea and improved egg production we previously reported in these birds [28].

## 2. Materials and Methods

### 2.1. Animal Trial and Bird Management

The study was approved by the Animal Ethics Committee of Central Queensland University under approval number 0000022879.

The study was performed on a commercial layer farm in Queensland, Australia. Specialised Breeders Australia P/L (Bendigo, Victoria) provided the Lohman-Brown day-old chicks. The chicks were brooded and reared on grower sheds until they reached 16 weeks of age with ad libitum access to feed and water. After the rearing stage, birds were randomised and transferred separately into the production shed, with 20,000 birds each in the treatment and control group with ad libitum access to water and feed. The birds in the treatment and control group had no physical contact indoors or in the range.

The birds were fed with a standard layer diet as an unsupplemented control (Ctr) on one side of the shed or with PFA supplemented feed (Phy) in the other section. The ratio of the feed ingredients was changed over time to adjust for bird age and production stage, but all the same main ingredients were used throughout the production cycle. The feed was provided in the form of a mash only. The phytogenic product used was the feed additive Biostrong^®^ Protect (Delacon Biotechnik GmbH, Engerwitzdorf, Austria). The PFA was supplemented at the recommended dose rate of 600 mg/kg. PFA blend comprised a mixture of essential oils from the *Myrtaceae* (myrtle family) and *Asteraceae* (daisy family) and saponins. The supplementation lasted from week 16 to week 40 of bird age. The birds were monitored for the next ten weeks until week 50. We aimed to evaluate the efficacy of PFA against Spotty Liver Disease common in this region of Australia during the hottest summer period. The supplementation period started just before the height of the summer in mid-September, and the sampling of the birds was at 30 weeks of age, aiming at the end of the peak of production stage and at the peak of summer heat, collected on 20th of December with the maximum daily temperature of 38 °C on the sampling day. Birds were randomly selected from different parts of the shed, rejecting the outlier birds.

Performance parameters measured regularly included the rate of lay (ROL, measured daily as eggs produced over the number of birds in the shed), cumulative mortality, body weight, feed conversion ratio (feed consumed per dozen of eggs), cumulative hen housed eggs (total number of eggs compared to the number of birds), GBD (grams of feed consumed per bird per day), egg weight, eggshell thickness, cumulative dirty eggs (rejected dirty eggs), yolk colour and Haugh units. Performance, microbiota and metagenomic functional analysis were separately presented in Yu et al., [28].

At 30 weeks of age, ten birds from each treatment were euthanised, and ileum tissue was collected for RNAseq analysis and histology. Ileum tissue for RNAseq was snap frozen in liquid nitrogen and stored at −80 °C, and for histology, it was stored in 10% neutral buffered formalin.

### 2.2. RNA Sequencing and Analysis

Around 100 mg of tissue samples were homogenised with 1 mL of TRIsure (Cat# BIO-38033, Bioline Meridian Bioscience, London, UK) by OMNI tissue homogeniser TH (OMNI International, Kennesaw, GA, USA) and centrifuged with phase separation by addition of 200 μL of chloroform. The Isolate II RNA Mini Kit (Bioline, CAT# BIO-52072) was used to clean up the RNA from the aqueous phase and isolate the RNA following the manufacturer’s instructions. This method was used previously in other similar studies to isolate good quality RNA [29,30,31,32]. Extracted RNA was freeze-dried and shipped to the sequencing facility (Azenta Life Sciences, Beijing, China) in RNAstable tubes. TruSeq RNA Library Prep Kit v2 #RS-122-2001 (Illumina, San Diego, CA, USA) was used to prepare the library, which was sequenced with Illumina Novaseq 600 platform with 2 × 150 bp paired-end configuration. Sequence data is available on the NCBI Sequence Read Archive (SRA) database with accession number PRJNA887923.

The alignment of the sequences with the chicken genome and transcriptome analysis was done using CLC Genomic Workbench 21.0.3 (Qiagen, Hilden, Germany). The differential genes were selected using the DESeq2 R package, and further pathway investigation was done using Ingenuity Pathway Analysis (QIAGEN IPA, version 76765844). Genes included in IPA analysis were DESeq2 *p* < 0.05, with an absolute fold change of 1.2.

### 2.3. Histology

The ileum tissue samples were fixed in 10% neutral buffered formalin and embedded on paraffin wax. The histological sections were obtained by cutting thin tissue sections with a Microtome (Leica RM2135) and stained with Periodic Acid Schiff and Alcian Blue (PASAB) staining. Slides were scanned using Panoptiq™ software (ViewsIQ Inc., Vancouver, BC, Canada) and Nikon Eclipse Ci-L Plus biological microscope, and morphometric analysis was done using QuPath v0.3.0 software. Villus height (µm), crypt depth (µm), and the number of goblet cells were measured from 10 villi in each slide and from five slides per group. Statistical analysis of the measurements was done using GrpahPad Prism v9.

## 3. Results

### 3.1. Sequencing Quality and Differential Analysis

Ten samples, 5 for each group, were successfully sequenced with a total of 489.26 million raw sequences, with the largest sample of 58.7 million and the lowest sequenced sample of 41.4 million sequences. The raw sequence QC report is given in Figure S1 of Supplementary File S1. The sequences were all 150 nt long, with no ambiguous sequences and average quality scores ranging from 20 to 38 (Supplementary File S1).

**Figure 1:**
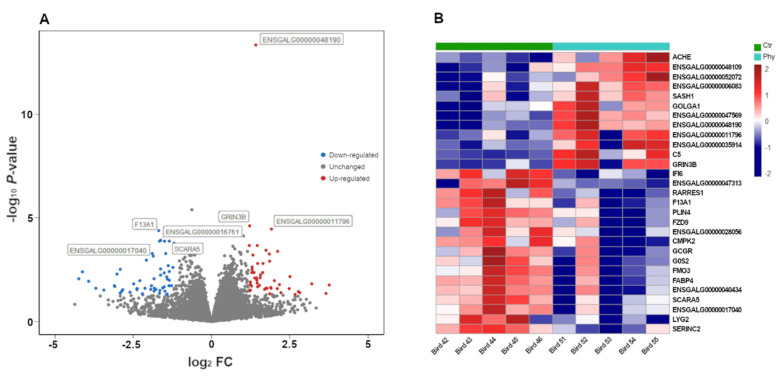
Effects of PFA supplementation on ileum gene expression. (**A**) Volcano plot of genes showing differentially expressed genes, red = upregulated, blue = downregulated, grey = unchanged. (**B**) Heatmap showing the most differentially expressed genes.

Out of 16,666 mapped and expressed genes, differential analysis done in DESeq2 revealed 202 genes with *p* *<* 0.01 and 720 with *p* *<* 0.05. A total of 505 genes were imported into IPA analysis with *p* < 0.05 and absolute fold change > 1.2. Figure 1 shows the most significantly altered genes, while Figure S2 of Supplementary File S1 shows the box plots of the most significantly altered genes.

### 3.2. Pathway Analysis

Ingenuity Pathway Analysis (IPA, Qiagen, Germany) is based on more than 10 million manually curated gene expression-based annotations in the Ingenuity Knowledge Base (IKB). This software is widely used to discover networks, interactions, and novel regulators related to the uploaded gene lists. We used the Canonical Pathways function in IPA to identify pathways significantly altered by PFA (*p* < 0.01) and differentiate if the pathway was activated or inhibited due to PFA supplementation using z-score statistics.

**Figure 2:**
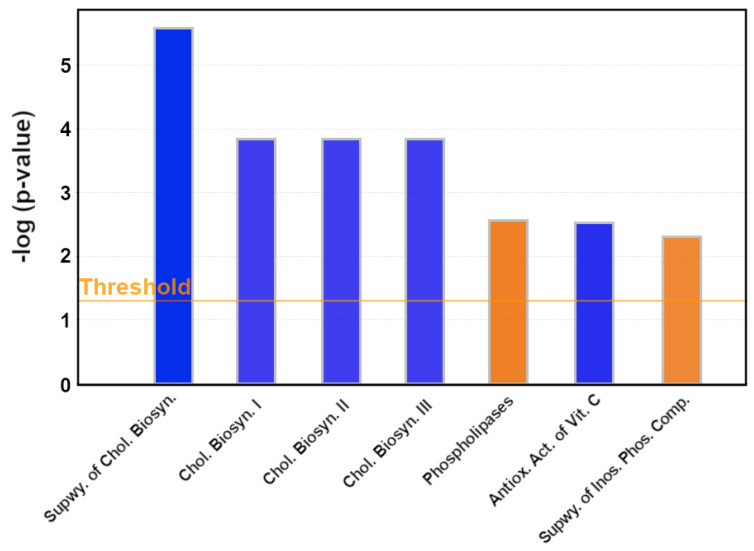
The most affected canonical pathways. The bars are coloured by z score, with blue indicating an inhibition and a negative z score (z < −2), and orange colour indicating activation (z > 2). The orange line shows −log (*p*-value) significance threshold of *p* = 0.05. An extended figure is provided in Supplementary File S1.

PFA significantly inhibited four cholesterol pathways, including the Super-pathway of Cholesterol Biosynthesis, Cholesterol Biosynthesis I, Cholesterol Biosynthesis II (via 24, 25-dihydrolanosterol), and Cholesterol Biosynthesis III (via Desmosterol) (Figure 2). The significance values for the canonical pathways were calculated using the right-tailed Fisher’s Exact Test to determine enrichment. The supporting data, including the altered gene list, is given in Table S1 of Supplementary File S1.

### 3.3. Upstream Analysis

The upstream analysis from IPA helps predict gene expression overlaps from the selected dataset with the IPK database. The upstream analysis identifies possible transcriptional regulators explaining investigated gene expression changes. The upstream regulators can be host transcription factors, but also any molecule that, based on the IPK expression database, has identical or reversed effects on a significant number of genes as the PFA treatment. The opposite, or reversed, effects on gene expression result in an inhibited regulator that can undo the effects of the treatment investigated, in this case, PFA. The identical transcriptional effects of the proposed regulator and PFA result in an activated regulator effect, suggesting that the regulator and PFA can enhance each other’s action. In addition to host transcription factors, these molecules can be gene regulators from other host species, enzymes, micro-RNA, receptors, chemicals, and drugs, as long as they target a significant number of altered genes in the analysed dataset. This helpful feature allows us to identify possible drug interactions, or if an emerging disease is investigated, this feature could identify a range of existing developed drugs that can undo at least some effects of the disease.

Based on the IPA analysis, 17 regulators were most significantly overlapping with PFA transcriptomic effects (*p* < 0.01) in the same direction and thus highly significantly activated (z > 2). They included l-asparaginase, prostaglandin E2 and beta-carotene, drugs nelfinavir, calcitriol, arsenic trioxide, chemical toxicants pyridaben and maneb, cytokine OSM, enzymes POR, FBXW7 and PRKCD, transmembrane receptors IL6R and ITGB4 and other molecules shown in Table S2 of Supplementary File S1. The supplementary file shows all regulators and the genes regulated by both PFA and the regulator. Among the most activated regulators, l-asparaginase, nelfinavir, calcitriol, OSM, PRKCD, KDM5B, arsenic trioxide, beta-carotene, WT1, TEAD4, vorinostat, slirasib, tanespimycin, and sirolimus can treat cancers by their antineoplastic activity, prevention of metastasis, and suppression of tumour cells (IPK database) suggesting that PFA has considerably similar clinically significant anticancer properties.

The IPA analysis reported 24 predicted inhibited gene expression regulators that have the significant opposite effect (*p* < 0.01, z < −2) on the gene expression compared to PFA, with the remaining regulators shown in Table S2 of Supplementary File S1. These include exogenous chemicals linoleic acid, elaidic acid and inosine, registered drugs ezetimibe, norephedrine, rosuvastatin, genistein, clozapine, pitavastatin, isoquercitrin, rosiglitazone, transcription factors SIRT2, SIRT1, KLF15, SREBF1, CTNNB1, SREBF2, ESRRA and a transmembrane receptor LGR4. A complete list is given in Table S2 of Supplementary File S1. According to IPA database annotations, among these regulators, there were molecules or transcription factors highly related to lipid metabolism; SREBF2, SREBF1, SCAP, AKT1, INSR and RETN.

Based on the upstream regulator analysis data, prolonged supplementation of the PFA can interfere with a range of medical drugs, either enhancing their effects by amplifying gene expression changes caused by the drug, or in other cases, inhibiting the effects of some drugs by having the opposite effect on the high number of genes altered by this drug (Table 1).

### 3.4. Regulatory Effects

**Figure 3:**
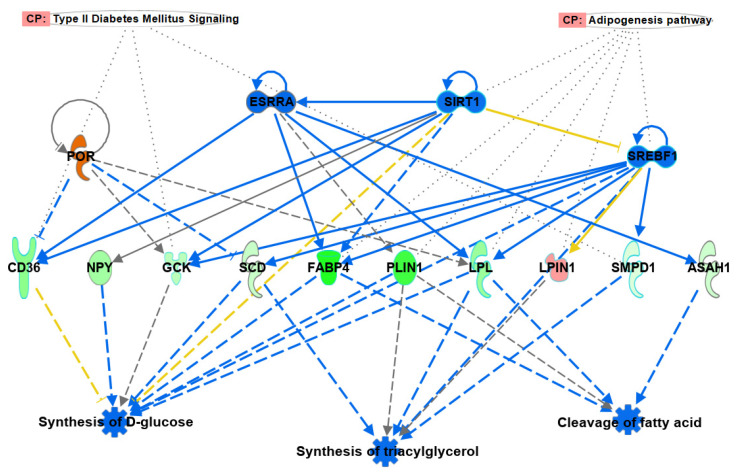
Chicken transcription factors predicted to control reduction in fat deposition and consequent reduction in type 2 diabetes signalling via alterations in glucose and fat metabolism.

The Regulator Effects analysis in IPA identified five predicted regulatory networks which combine multiple upstream regulators expressed in our dataset to identify the significant influence of their activation/inhibition in our dataset on the regulator-controlled disease and functions. The top-scoring network (Figure 3) shows how activation of POR and inhibition of ESRRA, SIRT1 and SREBF1 results in altered gene expression of 10 target genes, which affects the significant inhibition of synthesis of glucose, triglycerol and cleavage of fatty acids. This was tightly connected with the reduction of Type 2 diabetes signalling and the Adipogenesis pathway, contributing to reduced development and accumulation of adipose tissue (fat) across all body locations, both as subcutaneous fat and as depots.

### 3.5. Diseases and Functions

IPA’s diseases and functions feature allows us to predict whether the PFA can increase or reduce susceptibility to diseases or alter significant metabolic functions by comparing PFA-altered genes to the IPK database. Complete data is provided in Table S3 of Supplementary File S1 and sorted by function for clarity of interpretation in Table S4 of Supplementary File S1.

The analysis identified clear trends in both PFA inhibited and activated diseases and functions. Inhibited diseases and functions are dominated by major categories Cancer, Carbohydrate metabolism, Cardiovascular disease, Organismal injury and abnormalities, Developmental disorder, Inflammatory response, Lipid metabolism and Nutritional disease. Activated categories were dominated by Amino acid metabolism, Cardiovascular disease, Cell movement and survival, Connective tissue inflammation, Quantity of glucagon, Quantity of blood cells and leucocytes, RNA virus infection and Lipid metabolism.

**Figure 4:**
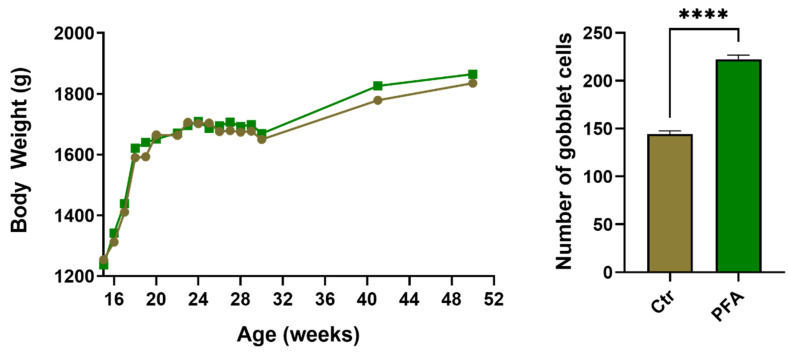
Bird body weight of the birds from the start of PFA supplementation and histological examination showing a significant increase in the number of ileum goblet cells (**** = *p* < 0.0001).

Categories with both activated and inhibited arms, such as 14 inhibited and five activated categories of Lipid metabolism, in combination would result in a faster turnover of steroids and triglycerols. In addition to this, seven inhibited categories of Carbohydrate metabolism would result in less glucose in the bloodstream; significantly altered and marginally activated Quantity of amino acids (*p* = 3.33 × 10^−7^), and a Quantity of glucagon (*p* = 0.00065), which is known to raise the concentration of glucose and fatty acids in the bloodstream all indicate major alterations of metabolism. In combination, all of these metabolic changes are predicted to result in reduced Weight gain and inhibited Obesity, which is among the most significantly inhibited categories, propelled by a faster turnover of glucose and fat and likely more muscle building. Cross-referencing this with the actual bird performance data (Table S3), the weight of the PFA-treated birds was consistently higher than the control from week 25 to the end of supplementation at 40 weeks (Figure 4) which persisted to the end of data monitoring at 50 weeks old Yu et al. [28].

Twenty six cancer categories were significantly inhibited, including multiple types of gastrointestinal and digestive system-related cancers, a range of haematological cancers of different types of leucocytes, and a range of genitourinary cancers, like pelvic and breast cancer. While other categories, like lipid and carbohydrate metabolism, had some arms inhibited and others activated, all affected cancer categories were inhibited by PFA.

Another group of inhibited categories includes the reduction of developmental abnormalities, with the overall Perinatal death category amongst the most significant and most inhibited (*p* = 1.96 × 10^−7^, z = −2.13). Significantly reduced Inflammatory response (*p* = 0.00033) and Inflammation of the body cavity (ileum) is in agreement with the significantly increased Quantity of goblet cells category (*p* = 5.73 × 10^−4^, z = 1.97), Tables S4 and S5 of the Supplementary File S1. This is in agreement with our histology data that showed no change in villi and crypt morphology but showed a significant increase (*p* < 0.0001) in the number of goblet cells (Figure 4).

**Figure 5:**
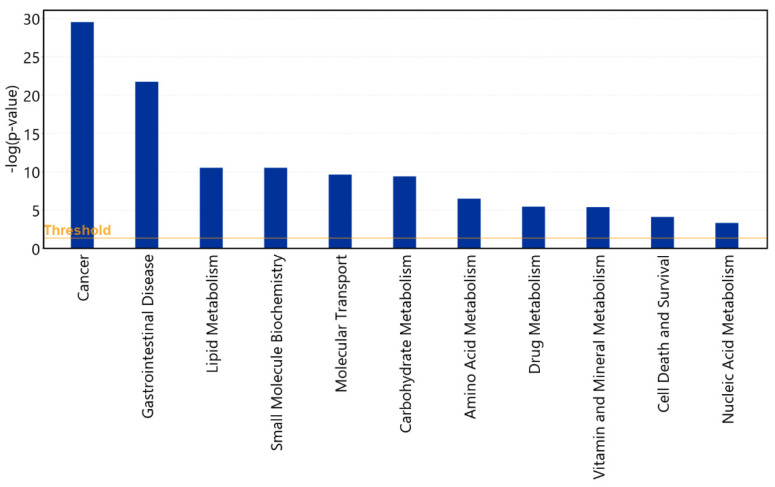
Major Disease and Function categories, most influenced by the supplementation of PFA. The PFA strongly inhibits a range of cancers and gastrointestinal diseases while altering the metabolism that mimics obesity reduction but practically results in heavier animals.

Hypothetically, based on the data from Supplementary Tables S4 and S5 and Figure 5, if we were to translate disease and function prediction findings to possible human use of this product, we would not recommend PFA to those suffering from seizures (increased by PFA, *p* = 7.32 × 10^−5^, z = 2.23), blood clots and formation of thrombus, those with issues with urination disorder or retroviral infections. We could recommend it to those with gastrointestinal inflammation, gastrointestinal and other cancers, obesity, pregnancy, and those who want to lose fat and gain muscle.

## 4. Discussion

Based on the upstream analysis, prolonged phytogenic supplementation demonstrated both activation and inhibition of the regulators specifically related to lipid metabolism and tumorigenesis. The synthesis of glucose and lipid biosynthesis are prominent metabolic processes associated with cancer metabolic reprogramming. This metabolic alteration is a prerequisite for the fast proliferation of cancer cells [33]. The Warburg effect, which is the increase in the use of aerobic glycolysis and glucose uptake, is the most significant metabolic alteration in the cancer [34], however, lipid metabolism also plays a vital role in the development of cancer cells. These cells harness lipid metabolism to acquire energy and cellular components for biological membranes, signalling molecules for survival, proliferation, metastasis, and invasion [35]. This may suggest that alterations in lipid and carbohydrate metabolism by PFA also play a role in predicted PFAs cancer-reducing capacity.

Two of the sterol regulatory element-binding transcription factors 1 and 2 (SREBF1 and SREBF2) were highly inhibited (z = −2.21 and −2.44) by the phytogenic supplementation. Together with SREBP-cleavage activating proteins (SCAP), they are crucial for maintaining cellular lipid homeostasis [36]; hence their aberrant activation is known to cause fatty liver disease, insulin resistance, obesity, and cancer development [37]. Obesity was also significantly decreased (*p* *=* 4.02 × 10^−6^, z = −1.13).

Similarly, nine categories of Lipid metabolisms were decreased in the Disease and Functional analysis. Notably, the metabolism and synthesis of sphingolipid (SL) were decreased. Sphingolipids with sphingosine-1-phosphate (S1P) and ceramide are important biomolecules with extensive effects on vital cellular processes, including programmed cell death, cell growth, and proliferation [38]. The perturbations of SL metabolism will change the responsible enzymatic pathways, which in turn can take part in the progression of various cancer cells such as colon [39] and breast cancer [40]. Nagahashi et al. (2016), through an in vitro experiment, demonstrated that cancer cells in breast cancer contain a higher amount of SL than in normal breast tissue [40]. Other lipid-related inhibited categories include Metabolism and Synthesis of triacylglycerol, Cleavage and Hydrolysis of fatty acids, Hydrolysis and Catabolism of lipid, and Lipolysis, which could have led to perturbation in the lipid metabolism preventing the tumour-adipose microenvironment. This is in agreement with phytogenic supplementation reducing the disease susceptibility for numerous cancer categories, including breast and colorectal cancers.

Various anticancer upstream regulators or drugs were activated (upstream analysis), suggesting that they have similar effects to PFA. The activated drug nelfinavir is the most potent anticancer drug inhibiting 60 cancer cell lines derived from nine different tumour types [41]. In in vivo experiment done by Gills, Lopiccolo and Dennis [41], nelfinavir inhibited tumour growth and upregulated markers of endoplasmic reticulum stress, apoptosis, and autophagy. This drug was clinically tested and used to treat patients with breast cancer [42], thyroid cancer [43], and renal cancer [44]. Similarly, vorinostat, an antineoplastic drug actively used in current medicine, was also activated. This is an inhibitor of class I and II of histone deacetylases [45], which have promising effects as anticancer agents inhibiting the proliferation of cancer cells in breast cancer [46], renal cancer [47], and pancreatic cancer [48]. Aside from drugs, calcitriol, the most active metabolite of vitamin D, was upregulated, which has significant antineoplastic activity in various cancers by regulating multiple signalling pathways, including inhibition of proliferation associated with cell cycle arrest, induction of apoptosis, and reduction in invasiveness and angiogenesis [49]. Beta-carotene, a natural nutrient widely found in vegetables and fruits and known to be beneficial to human health, was also activated. Like the phytogenic products, beta-carotene has a clear role in antioxidant, immune-related functions, and cell gap junction-related functions [50] and has been explored by many researchers to treat gastric cancers [51] and to decrease the proliferation of colon [52] and prostate cancer cells [53].

There were several other less explored molecules or regulators with anticancer properties upregulated by PFA in this study. Although plant-derived natural products have demonstrated outstanding anticancer properties to effectively suppress early and intermediate stages of carcinogenesis in their pure form [17,54,55], their pharmacological potential is impeded by their poor penetration into cells, poor bioavailability, narrow therapeutic index, rapid uptake by normal tissues, and low aqueous solubility [56]. To alleviate this problem, synergistic studies on facilitating nanoparticles have shown improvements in treating cancer by greater stability and solubility of phytogenic compounds with enhanced tissue targeting and cellular uptake [56].

An increase in Weight loss (*p* = 7.26 × 10^−5^, z = 2.499) and a decrease in Weight gain (*p* *=* 6.70 × 10^−6^, z = −2.007) were among the most affected annotations in the category of Nutritional disease. There were 15 of 20 genes with expression change direction consistent with an increase in Weight loss, including the *DGAT2* genes. Diacylglycerol O-Acyltransferase 2 (DGAT2) is a critical catalysing enzyme for triglyceride (TG) biosynthesis and storing fat [57], resulting in obesity in humans [58]. *DGAT2* is an essential marker gene to trace fat deposition for meat processing animals, and numerous studies have been done to elucidate the relationship between intramuscular fat content and meat quality [59,60,61,62]. Recently, a positive correlation of *DGAT2* genes with obesity has been reported [58].

Intriguingly, although several genes related to weight gain were downregulated in phytogen-treated birds, these birds were heavier than the birds on control diet (Figure 4). As presented and discussed above, lipid metabolism was affected by the inhibition of several functional categories, including Metabolism and Synthesis of triacylglycerol, which is the form in which fat is stored in the body. Therefore, a reduction in Obesity or Weight loss categories is most likely the result of less fat storage. Notably, the category Quantity of amino acid was significantly increased (*p* = 3.33 × 10^−7^, z = 1.187) in the PFA-supplemented group with upregulation of *SLC6A18* gene, which is the epithelial neutral amino acid transporter. This indicates that the higher body weight of PFA-supplemented birds compared to control birds was not due to an increase in fat but to an increase in lean muscle.

Results from the histology data (Figure 4) in conjunction with transcriptomic analysis have demonstrated a tremendous increase in the number of goblet cells in PFA-supplemented birds. Goblet cells secrete mucin responsible for generating the mucus layer to protect the gut against pathogen invasion [63]. Mucins are composed of highly hydrophilic gel-like glycoproteins that help prevent direct contact with pathogenic microorganisms [63]. A reduced amount of mucin layer could allow intestinal mucosal barrier dysfunction [64], causing an excessive immune response in the host and resulting in colitis [65] and diarrhoea [66]. This is in agreement with the lower amount of dirty eggs produced in the PFA-supplemented birds in the previous study [28] as dirty eggs are an indicator of diarrhoea and intestinal issues in birds. This could have been contributing factor in the improved performance of PFA-supplemented birds during the Spotty Liver Disease outbreak in this trial. Increasing goblet cells promotes a healthier intestinal mucosal layer and is ideal for maintaining gut immune homeostasis.

## 5. Conclusions

The effects of this PFA blend on ileum gene expression show substantial benefits on the gastrointestinal system. It is also beneficial for controlling high blood pressure, obesity, diabetes, atherosclerosis, and cholesterol levels. This PFA can cause weight loss by altering the ratio of fat and carbohydrate metabolism toward lower fat storage. This could be a useful feature in the pig finisher growth stage. An increase in the number of goblet cells reduced inflammation levels in the ileum, suggested by significantly decreased Inflammation of body cavity disease category, and by a reduced number of dirty eggs.

Based on predicted disease profiles, this PFA would be beneficial for overall intestinal health but should be avoided in flocks infected with poultry retroviral infections: the avian leukosis/sarcoma virus (ALSV), reticuloendotheliosis virus (REV), and lymphoproliferative disease virus (LPDV) of turkeys. Transcriptomic data allows us to understand overall effects, including other indications of when it would be beneficial to use or avoid the product. This type of comprehensive mechanistic research investigation is a step toward a shed personalised treatment selection based on the burning issues in each farm and production system. A better understanding of PFAs’ actions can maximise their benefits and make the first steps toward the customised farm, shed or production system supplementation.

## Figures and Tables

**Table 1:** IPA upstream analysis identified possible PFA interference with known medical drugs. Drugs shown in the pink background are activated, and those in blue are inhibited by PFA. Descriptions were taken from the IPK database.

Drug	Description (IPK Database)
Vorinostat	Anticancer drug
Salirasib	Anticancer drug
Tanespimycin	Anticancer drug
Arsenic trioxide	Anticancer drug
Sirolimus	Anticancer drug; Immunosuppressant
Dimethyl sulfoxide	Anti-inflammatory agent
Nelfinavir	Antiviral drug
Calcitriol	Calcium regulator
Genistein	Angiogenesis inhibitor
Isoquercitrin	Anti-cachexia drug
Streptozocin	Anticancer drug
Methylprednisolone	Anti-inflammatory and immunosuppressive medication.
Norephedrine	Direct agonist of adrenergic receptors
17-alpha-ethinylestradiol	Estrogen used widely in birth control pills
Clozapine	Psychiatric medication, antipsychotic (schizophrenia)
Rosuvastatin	Statin medication used to prevent cardiovascular disease and treat abnormal lipids
Rosiglitazone	The antidiabetic drug, works as an insulin sensitiser
Pitavastatin	For high blood cholesterol
Clofibrate	For high blood cholesterol
Cerivastatin	For high blood cholesterol
Ezetimibe	For high blood cholesterol
Lovastatin	For high blood cholesterol

## Data Availability

All sequencing data are publicly available in NCBI SRA database under project number PRJNA887923 (https://www.ncbi.nlm.nih.gov/bioproject/887923).

## Supplementary Materials

The following supporting information can be downloaded at: https://www.mdpi.com/article/10.3390/antibiotics11101428/s1, Table S1: Differentially abundant pathways, Table S2: Upstream analysis results, Table S3: Bird performance data, Table S4: Significantly affected disease categories, Table S5: Significantly affected disease categories sorted by disease, Figure S1: Sequencing report for raw sequences per sample, Figure S2: Top differentially expressed genes
